# The 30-year evolution of motor vehicle road injuries: Can the future come from the shadows?

**DOI:** 10.1371/journal.pone.0342257

**Published:** 2026-03-03

**Authors:** Songxiahe Zhao, Zhongjiang Lan, Jinrui Lin, Shihu Kan, Yanliang Jiao, Lei Chen, Yibin Du

**Affiliations:** 1 Department of Orthopedics, The Third Affiliated Hospital of Anhui Medical University (The First People’s Hospital of Hefei), Hefei, Anhui, China; 2 Graduate School of Anhui Medical University, Anhui Medical University, Hefei, Anhui, China; 3 Department of Intensive Care Medicine, The Second Affiliated Hospital of Anhui Medical University, Hefei, Anhui, China; Creighton University, UNITED STATES OF AMERICA

## Abstract

**Background:**

This study examines temporal changes from 1990 to 2021 in the burden of vertebral fractures (VFs) attributable to motor vehicle road injuries (MVRIs), with a particular focus on age- and sex-specific patterns in China and India. These national trends are compared with global patterns to better understand population distribution characteristics and injury mechanisms underlying this public health challenge.

**Methods:**

Data were obtained from the 2021 Global Burden of Disease (GBD) study. Crude rates and age-standardized rates (ASRs) of incidence, prevalence, and years lived with disability (YLDs) for MVRI-related VFs were estimated. Joinpoint regression was applied to assess temporal trends, while age–period–cohort (APC) modeling was used to disentangle the independent effects of age, calendar period, and birth cohort.

**Results:**

From 1990 to 2021, the global age-standardized incidence rate (ASIR) of MVRI-related VFs declined by 48.8%, with an average annual percentage change (AAPC) of −1.839% (95% confidence interval [CI], −1.869 to −1.808). In contrast, China showed no significant reduction in ASIR (AAPC = −0.478%, 95% CI, −0.531 to −0.426), whereas India demonstrated minimal variation over the study period (AAPC = −0.013%, 95% CI, −0.054 to 0.028). Regional analyses revealed heterogeneous drivers of disease burden. In China, period effects during 2000–2021 were strongly associated with elevated risk among males aged 20–40 years, likely reflecting hazardous driving behaviors, while cohort effects were most prominent among individuals born between 1980 and 1990. Conversely, individuals older than 60 years experienced an increasing burden, potentially related to osteoporosis and rapid motorization. Across all regions, males consistently exhibited higher ASIRs, age-standardized prevalence rates (ASPRs), and YLD rates than females, with the greatest sex disparities observed among younger males in China.

**Conclusion:**

The persistently high burden of MVRI-related VFs in China, which diverges from declining trends observed in countries with a high sociodemographic index (SDI), highlights the need for targeted prevention strategies. Interventions should prioritize behavioral risk reduction in younger male populations and address age-related biomechanical vulnerability in older adults. In India, strengthening road safety enforcement and trauma care infrastructure remains essential. These findings underscore the heterogeneous demands for road injury prevention in China and India and provide evidence to support more effective allocation of public health resources.

## Introduction

Motor vehicle road injuries (MVRI) remain a major global public health challenge and significantly contribute to disability and mortality [1,2]. Traffic accidents are a major type of high-energy trauma, with 50 million nonfatal injuries and 1.3 million deaths occurring on an annual basis, which consequently represent the eighth leading cause of death worldwide according to Global Road Safety Monitor data [3]. Vertebral fractures (VFs) represent a key but often overlooked consequence of MVRI, with complex demographic and biomechanical implications being reported. Although prior studies have reported global declines in MVRI burden, it remains unclear as to how these trends differ across countries at different development levels, particularly in rapidly motorizing middle-income countries such as China and India [4,5]. For those who sustain concurrent spinal cord injuries during the traumatic event, outcomes are further worsened by irreversible neurological damage and high-risk complications, including respiratory failure [6]. The aim of this study is to compare the long-term trajectories of MVRI-related VFs in China, India, and throughout the world, thereby identifying heterogeneous epidemiological patterns and informing context-specific prevention strategies [7].

The global epidemiological distribution of MVRI-precipitated VFs exhibits significant regional variability with disparities that are influenced by geographic and sociodemographic factors. Between 1990 and 2021, high-income regions achieved sustained reductions in both incidence and absolute cases through innovations such as electronic stability control and smart traffic systems, and this trend is exemplified by the Vision Zero initiative of Sweden. However, socioeconomic disparities persist, with low- and middle-income regions lagging in both the implementation of preventive infrastructure and access to postinjury care [8]. For example, China, which is currently in a phase of rapid economic transition, has experienced a decrease in incidence but an increase in the number of cases, thus indicating a distinct epidemiological trajectory that has demonstrated China as a global hub for VFs caused by MVRI [3,9].

From 1990 to 2021, rapid industrialization, urbanization, and motorization drove significant changes in the age- and sex-specific patterns of motor vehicle-related VFs. Pediatric groups achieved improved outcomes owing to strengthened social protection, whereas young adult males, who disproportionately engage in risky driving behaviors, remained a high-risk group [10]. Moreover, aging populations, which face the dual challenges of demographic aging and the progression of osteoporosis, have shown increasing rates of fracture-related disability [1]. In summary, more precise and varied strategies are needed to reduce the burden of disease for people of different ages and genders.

Prior studies faced three limitations: limited scope, small cohorts, and methods that could not distinguish age/period/cohort effects, which hindered effective interventions. Using data from the Global Burden of Disease 2021, we applied Joinpoint regression and Bayesian modeling to analyze and compare the trends in the incidence, prevalence, and years lived with disability (YLDs) associated with motor vehicle-related vision loss in China, India, and globally. Our findings identify modifiable risks and population-specific vulnerabilities and provide evidence for tailored prevention strategies and optimized rehabilitation resource allocation.

## Materials and methods

### Data sources and processing

The epidemiological metrics of VFs were derived from the Global Burden of Disease (GBD) 2021 database (https://vizhub.healthdata.org/gbd-result/), which was accessed on December 6, 2024. This dataset covers 71 diseases and injuries across 204 countries and quantifies 88 risk factors with 95% uncertainty intervals (UIs) [11,12].The following steps are crucial for obtaining the required data: open the GBD 2021 database, select the “Injury by Nature” option in the “GBD Estimate” column, select the “Spinal Fracture” option in the “Injury” column, select the “Motor Vehicle Road Injuries” option in the “Cause” column, select the “Global, China, India, High SDI, High-Middle SDI, Middle SDI, Low-Middle SDI, Low SDI” options in the “Location” column, and then select the remaining options. Finally, download the data. This way, we can obtain the VFs data caused by MVRI and systematically exclude fractures caused by other types of trauma. In China, data on VFs are derived from multiple sources, including the China Disease Surveillance Points, disability registries, emergency department records, and the China Injury and Risk Factor Surveillance system (https://ghdx.healthdata.org/gbd-2021/data-input-sources). Empirical validation using analyses of China’s heterogeneous health datasets confirms the methodological rigor and epidemiological representativeness of the GBD database and reinforces its utility in identifying injury dynamics within complex healthcare frameworks. Stratification by sex and 5-year age cohorts was performed, and ASR was calibrated using the GBD 2021 global reference population to mitigate confounding from demographic heterogeneity. Disease classification adhered to the International Classification of Diseases (ICD-9, ICD-10) coding frameworks.

### Incidence, prevalence, YLDs, and standardized rates

VFs incidence was defined as the annual number of new motor vehicle-related fracture cases (1990–2021) and was calculated by dividing case counts by the mid-year population. Prevalence reflected active proportions of VFs across time and regions (China/India/global). Disability burden was measured via years lived with disability (YLDs) and incorporated temporary/permanent impairments by using GBD disability weights and duration models [11,13,14]. Age-standardized rates (ASIR/ASPR/ASYR) were derived via direct standardization against GBD reference populations to control for demographic confounders.

This secondary analysis used anonymized GBD data (https://vizhub.healthdata.org/gbd-result/). No human subjects were involved in the study design or implementation. In accordance with the Helsinki Declaration Article 32, ethics approval was waived for this retrospective analysis of deidentified aggregate data.

### Data curation and standardization

MVRI has emerged as a substantial epidemiological concern, particularly among younger demographics, for whom it disproportionately contributes to VFs—a critical yet understudied outcome compared with falls or osteoporosis-related fractures [15]. The GBD 2021 dataset enables granular analysis through its hierarchical stratification with raw data initially processed in Microsoft Excel 2021 using a dual-entry validation protocol to ensure fidelity. Missing values were imputed via GBD-default algorithms, whereas outliers underwent manual adjudication. The final datasets were structured into 5-year age‒period‒cohort matrices (1990–2021) to facilitate advanced modeling [1 3].

### Joinpoint regression analysis

Temporal trends were examined using Joinpoint Regression Software (v5.1.0; National Cancer Institute) to identify inflection points in ASIR, ASPR, and ASYR via Monte Carlo permutation testing (10,000 iterations) [16,17]. The annual percentage change (APC) was used to quantify segment-specific trends, whereas the average APC (AAPC) was used to characterize overall trajectories, with statistical significance determined at p < 0.05. Directionality (increase/decrease) was inferred by comparing APC/AAPC against a null trajectory.

### Age-period-cohort (APC) modeling

The DisMod-MR 2.1 Bayesian framework was used to disentangle age, period, and cohort effects on disease burden using Markov chain Monte Carlo (MCMC) algorithms [12,18]. Age effects reflect cumulative biological risk across the lifespan, period effects capture temporal influences (e.g., road safety policies), and cohort effects denote generational disparities in risk exposure. Model convergence was verified using Gelman‒Rubin diagnostics (potential scale reduction factor <1.1).

### Statistical analysis

Standardized rates were computed via the WHO 2000–2025 population weights. Spatiotemporal visualization and statistical inference were conducted in the R statistical environment (v.4.4.2) supplemented by *ggplot2* for cartographic outputs. APC-derived risk ratios and 95% confidence intervals were estimated to identify age‒period‒cohort interactions, with p < 0.05 indicating significance.

## Results

### Disease burden of VFs due to MVRI in China, India, and throughout the world

The total number of new cases in China exhibited a discernible shift from 60,807 (95% UI: 38,445–105,335) to 67,089 during the study period. Notably, the percentage of male cases increased from 66.8% (40,604 cases) to 72.4% (48,561 cases), whereas the percentage of female cases decreased from 33.2% (20,203 cases) to 27.6% (18,529 cases) (Table 1). The age-standardized incidence rate (ASIR) demonstrated a 14.1% reduction, decreasing from 5.04 to 4.33 per 100,000 population (95% UI: 2.49–6.80). Stratified by gender, the male ASIR decreased marginally from 6.41 to 6.11 per 100,000 (95% UI: 3.64–9.38), whereas the female ASIR plummeted by 31.9%, from 3.57 to 2.43 per 100,000 (95% UI: 1.24–4.27). Consequently, the male-to-female ratio increased from 1.80 to 2.51 (p < 0.001) (Table 1). Concurrently, China had a 5.8% decline in the age-standardized prevalence rate (ASPR), which decreased from 2.24 to 2.11 per 100,000, as well as an 8.3% reduction in the YLDs rate from 0.24 to 0.22 per 100,000 (Table 1).

**Table 1:** Age-standardized and all-age incidence, prevalence, and YLDs rates of VFs attributable to MVRI in China, India, and globally for 1990 and 2021.

	Year2021				Year1990	
Region	Measure	Gender	Numbers (95% UI)	Age-standardised rates per 100 000 (95% UI)	Numbers (95% UI)	Age-standardised rates per 100 000 (95% UI)
China	Incidence	Both	67089 (38445,105335)	4.33 (2.49,6.8)	60807 (33967,97828)	5.04 (2.81,8.12)
	Incidence	Male	48561 (28594,74850)	6.11 (3.64,9.38)	40604 (23705,63842)	6.41 (3.74,10.01)
	Incidence	Female	18529 (9807,32403)	2.43 (1.24,4.27)	20203 (10275,36334)	3.57 (1.83,6.33)
	Prevalence	Both	38615 (30709,49342)	2.11 (1.64,2.75)	23875 (17500,32559)	2.24 (1.68,2.97)
	Prevalence	Male	25947 (20332,33181)	2.86 (2.19,3.73)	15321 (11169,21093)	2.78 (2.1,3.71)
	Prevalence	Female	12668 (9965,16299)	1.35 (1.04,1.78)	8554 (6073,11926)	1.68 (1.23,2.3)
	YLDs	Both	4053 (2546,6082)	0.22 (0.14,0.35)	2566 (1498,4128)	0.24 (0.14,0.37)
	YLDs	Male	2745 (1703,4187)	0.3 (0.18,0.47)	1652 (964,2664)	0.3 (0.18,0.47)
	YLDs	Female	1308 (828,1931)	0.14 (0.09,0.22)	914 (529,1472)	0.18 (0.11,0.28)
Global	Incidence	Both	521907 (337585,764806)	6.46 (4.19,9.45)	609743 (389585,892396)	11.47 (7.38,16.74)
	Incidence	Male	352935 (233456,505250)	8.69 (5.75,12.46)	393088 (261277,557293)	14.69 (9.82,20.8)
	Incidence	Female	168972 (100249,263474)	4.21 (2.49,6.57)	216655 (126416,331330)	8.22 (4.82,12.58)
	Prevalence	Both	396377 (333899,472222)	4.72 (3.98,5.65)	441857 (367536,529013)	9.97 (8.36,11.64)
	Prevalence	Male	242715 (202819,292481)	6.01 (5.04,7.21)	262449 (216639,318225)	12.19 (10.3,14.35)
	Prevalence	Female	153661 (129688,179906)	3.51 (2.94,4.14)	179409 (148570,211479)	7.88 (6.64,9.17)
	YLDs	Both	40825 (26561,58080)	0.49 (0.32,0.69)	46042 (30012,65998)	1.03 (0.67,1.47)
	YLDs	Male	25207 (16363,36067)	0.62 (0.4,0.89)	27541 (17991,39479)	1.26 (0.82,1.79)
	YLDs	Female	15618 (10308,22201)	0.36 (0.24,0.51)	18501 (12169,26462)	0.81 (0.53,1.15)
India	Incidence	Both	46751 (28077,73050)	3.22 (1.95,4.99)	26379 (15212,40755)	3.22 (1.88,4.98)
	Incidence	Male	32736 (20113,49814)	4.34 (2.69,6.56)	16831 (10207,25344)	3.98 (2.46,6.03)
	Incidence	Female	14015 (7774,23622)	2.05 (1.15,3.43)	9547 (5159,15709)	2.4 (1.32,3.95)
	Prevalence	Both	20746 (15898,27545)	1.56 (1.22,2.04)	9972 (7327,13871)	1.53 (1.18,2.02)
	Prevalence	Male	13501 (10189,17951)	1.98 (1.54,2.57)	6210 (4586,8597)	1.81 (1.4,2.39)
	Prevalence	Female	7245 (5609,9502)	1.13 (0.88,1.46)	3762 (2678,5296)	1.22 (0.95,1.61)
	YLDs	Both	2183 (1326,3345)	0.16 (0.1,0.25)	1063 (619,1681)	0.16 (0.1,0.24)
	YLDs	Male	1433 (869,2234)	0.21 (0.13,0.32)	664 (390,1062)	0.19 (0.12,0.29)
	YLDs	Female	749 (467,1162)	0.12 (0.07,0.18)	398 (231,640)	0.13 (0.08,0.19)

Globally, the total number of incident cases of MVRI-related VFs declined markedly from 609,743–521,907 (95% UI: 337,585–764,806) accompanied by a 43.7% reduction in ASIR from 11.47 to 6.46 per 100,000 (95% UI: 7.38–16.74 vs. 4.19–9.45) (Table 1). Both male and female incidence rates exhibited statistically significant downward trends. The global ASPR decreased by 52.7% (9.97 to 4.72 per 100,000), whereas the YLDs rate decreased by 52.4% (1.03 to 0.49 per 100,000) (Table 1). In contrast, India experienced an increase in total new cases from 26,379–46,751 (95% UI: 28,077–73,050), although the ASIR, ASPR, and YLDs rate of India remained stable throughout this time period (Table 1).

### The incidence rates, prevalence rates, and age-standardized rates of VFs due to MVRI in different age groups in China between 1990 and 2021

**Fig 1:**
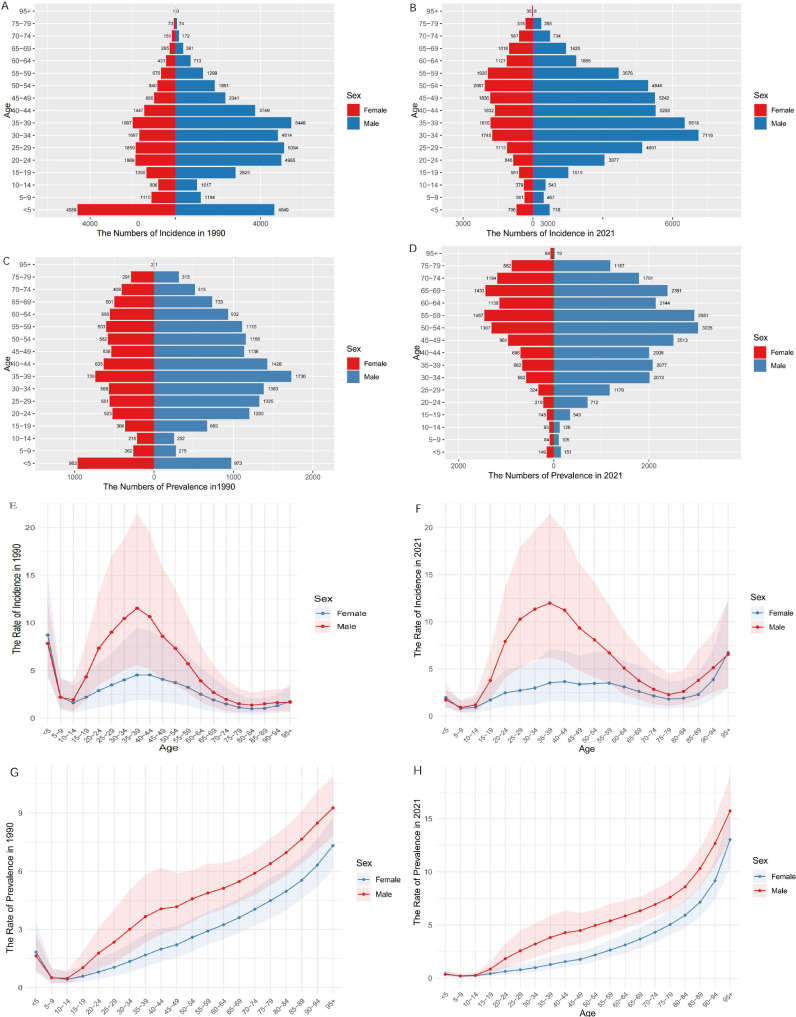
Age-specific numbers and age-standardized incidence and prevalence rates of MVRI leading to VFs in China in 1990 and 2021. (A) Age-specific incidence numbers in 1990. (B) Age-specific incidence numbers in 2021. (C) Age-specific prevalence numbers in 1990. (D) Age-specific prevalence numbers in 2021. (E) Age-standardized incidence rates in 1990. (F) Age-standardized incidence rates in 2021. (G) Age-standardized prevalence rates in 1990. (H) Age-standardized prevalence rates in 2021.

The age structure in 2021 changed compared with 1990. The most significant decreases in incidence and age-standardized rates were observed among children aged 0–5 years (Fig 1B, 1F), and a secondary upward trend was observed in people over the age of 75 (Fig 1F). Older populations also showed a second increasing trend (Fig 1F). The peak ASIR for males was unchanged from 35–39 years of age (Figs 1E, 1F), but was progressively younger with regard to the number of incidence cases (Figs 1A, 1B). The peak ASIR in females changed from 35–44 years to 40–59 years (Figs 1E, 1F), with gradual aging in terms of the number of incidence cases (Figs 1A, 1B). The trend of prevalence revealed that the highest prevalence rate was found among people aged 35–39 in 1990 (Fig 1C) and at 50–59 in 2021 (Fig 1D). The trend of ASPR with age generally increased for both males and females (Figs 1G, 1H). Notably, the ASIR, ASPR, incidence and prevalence were consistently greater in males than in females. The age-dependent trends in ASIR and ASPR in India, as well as the incidence and prevalence of different genders throughout the world, were similar to those reported in China (S1 Fig and S2 Fig).

### The burden of VFs caused by MVRI across different age groups in China, India, and throughout the world in 1990 and 2021

**Fig 2:**
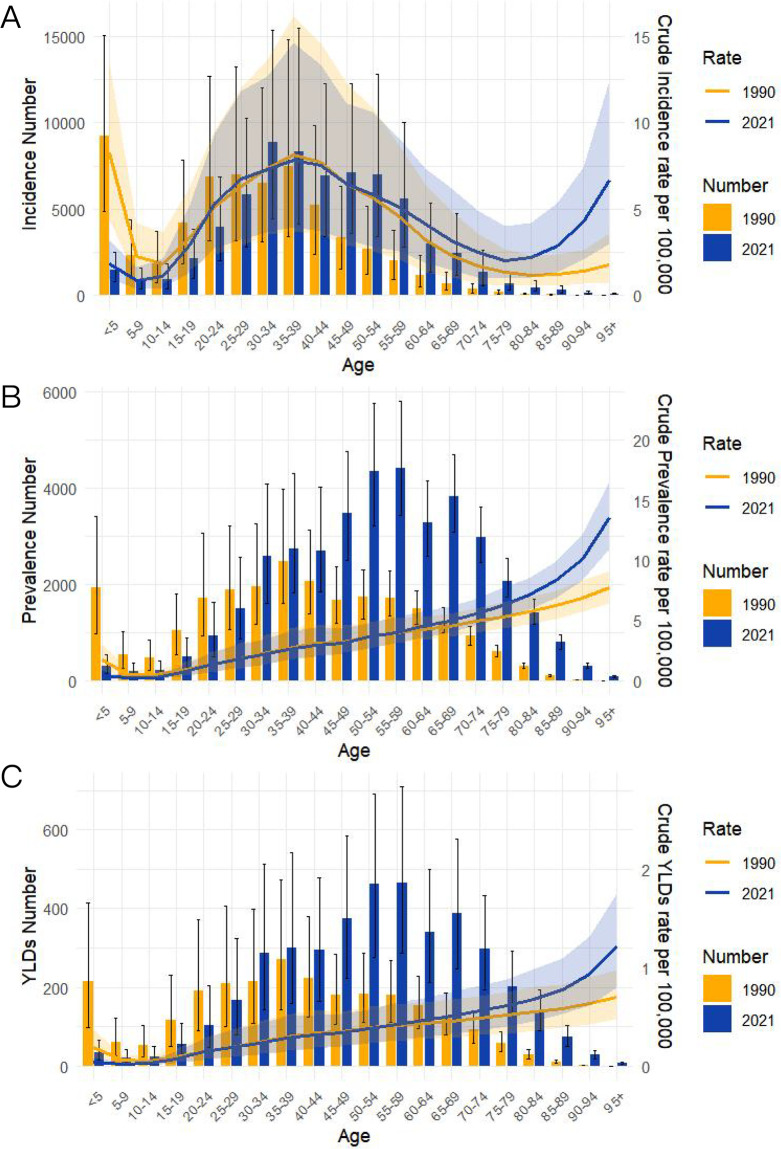
Trends in All-Age Cases and Age-Standardized Incidence, Prevalence, and Years Lived with Disability (YLDs) Rate for VFs by Sex in China (1990-2021). (A) Number and Rates of Incidence. (B) Number and Rates of Prevalence. (C) Number and Rates of YLDs.

Fig 2 depicts the trends of the crude incidence rate (CIR), crude prevalence rate (CPR) and crude YLDs rate (CYR) of VFs due to MVRI in China in 1990 and 2021. Compared with 1990, there was a significant increase in the number of new cases of CIR in 2021, with the 30–39 years age group representing the largest proportion (Fig 2A). Age stratification revealed that CIR declined most significantly in the 0- to 5-year age group, remained relatively stable in the 20- to 50-year age group, and increased significantly in the > 50-year age group (Fig 2A). CPR followed a similar trend as CYR with a decreasing trend in the < 15-year age group and an increasing trend in the > 60-year age group (Figs 2B and 2C). The total number of cases and the total number of YLDs continued to fluctuate at a high level, with the highest proportion being observed in the 50- to 59-year age group. Although China had a significant increase in new cases in 2021 compared with 1990, CIR, CPR and CYR decreased slightly. During the same period, India had trends similar to those of China, although the global trends of the indicators differed to some extent from China. Global CIR, CPR and CYR rates experienced a synergistic and significant decline with the total number of cases, whereas India showed a slight upward trend in the crude rates and a substantial increase in the total number of cases (S3 Fig and S4 Fig).

### Burden of vertebral fractures (VFs) linked to motor vehicle road injuries (MVRI) across SDI regions

**Fig 3:**
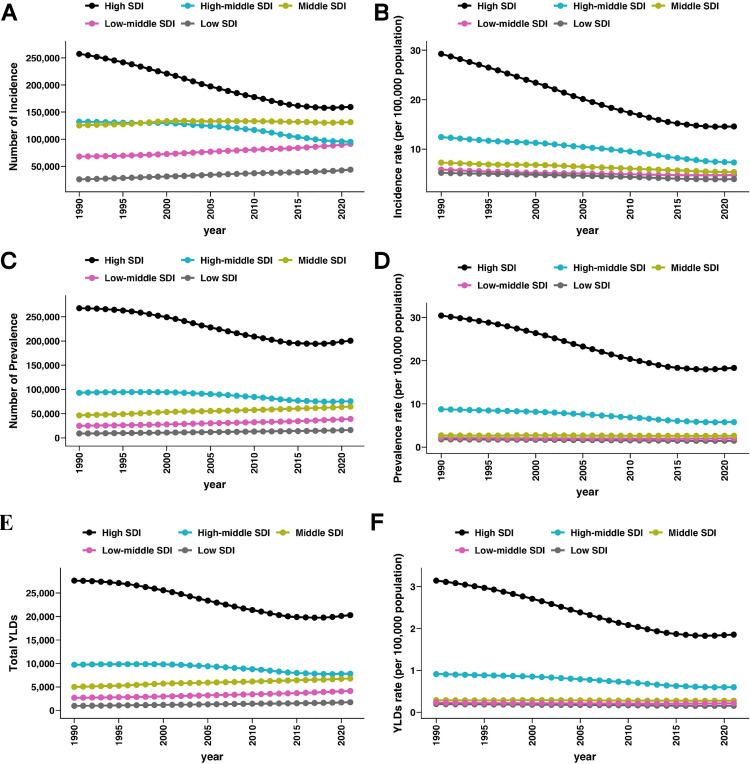
Trends in vertebral fractures (VFs) linked to motor vehicle road injuries (MVRI) across SDI regions (1990–2021). (A) Number of incident cases. (B) Incidence rates (per 100,000 population). (C) Number of prevalent cases. (D) Prevalence rates (per 100,000 population). (E) Total years lived with disability (YLDs). (F) YLD rates (per 100,000 population). Trends are stratified by the following sociodemographic index (SDI) regions: high, high-middle, middle, low-middle, and low SDI.

To further contextualize the burden of VFs linked to MVRI, we analyzed incidence, prevalence, and disability burden across sociodemographic index (SDI) regions in 2021. As shown in Fig 3A-B, the incidence rate was highest in high-SDI regions (14.6 per 100,000 population, 95% UI: 9.6–20.7), followed by high-middle (7.3, 95% UI: 4.7–10.9) and middle SDI regions (5.4, 95% UI: 3.3–8.1), whereas low-middle (4.8, 95% UI: 3.0–7.1) and low-SDI regions (3.9, 95% UI: 2.5–5.8) demonstrated substantially lower incidence. Prevalence patterns (Fig 3C-D) were similar, with high-SDI regions demonstrating the largest burden (18.3 per 100,000, 95% UI: 15.6–21.3), compared with high-middle (5.8, 95% UI: 4.9–6.9), middle (2.6, 95% UI: 2.1–3.4), low-middle (2.0, 95% UI: 1.6–2.7), and low-SDI regions (1.5, 95% UI: 1.1–2.0). In terms of disability burden (Fig 3E-F), high-SDI regions again demonstrated the most considerable load, with an age-standardized YLDs rate of 1.85 per 100,000 (95% UI: 1.23–2.61), which is approximately three times that of high-middle regions (0.60, 95% UI: 0.39–0.85) and markedly higher than middle (0.28, 95% UI: 0.17–0.42), low-middle (0.22, 95% UI: 0.13–0.33), and low-SDI regions (0.16, 95% UI: 0.09–0.25). Collectively, these findings indicate that although high-SDI regions have a greater absolute burden of VFs cases linked to MVRI, they also achieved more substantial long-term reductions, whereas lower-SDI regions (despite smaller absolute burdens being reported) demonstrated limited progress in reducing both incidence and disability, thus highlighting persistent disparities and the need for targeted prevention and rehabilitation strategies.

### Joinpoint regression analysis of the ASIR, ASPR and ASYR of VFs caused by MVRI in China, India and throughout the world

**Fig 4:**
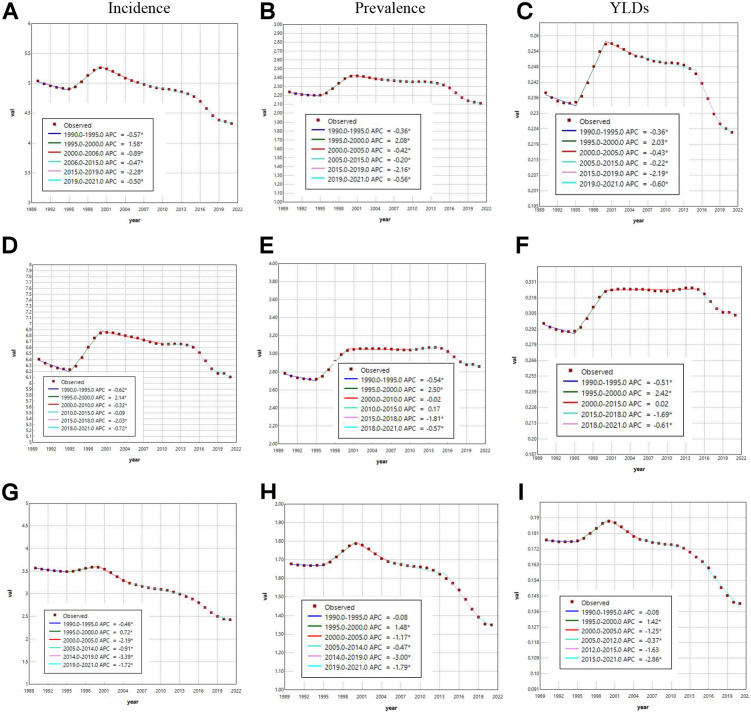
In China from 1990 to 2021, Joinpoint regression analysis was applied to the ASIR, ASPR and ASYR of VFs due to MVRI among males, females and both sexes, with a significance level of p < 0.05.

Fig 4 (which uses Joinpoint regression analysis) presents the dynamic trends and gender disparities in age-standardized indicators (ASIR, ASPR, and ASYR) of VFs caused by road traffic injuries in China from 1990 to 2021. Males presented a significantly greater disease burden across all metrics. The joinpoint analysis revealed a fluctuating pattern of rising and declining in China’s ASIR with turning points in 1995, 2000, 2006, 2015, and 2019. A pronounced upward trajectory was observed during 1995–2000 (Fig 4A) in which the male population demonstrated a steeper annual percentage change (APC: + 2.14, Fig 4D) than the female population did (APC: + 0.72, Fig 4G). The subsequent intervals predominantly exhibited declining trends with the most notable reductions occurring in males (2015–2018 APC = −2.03, Fig 4D) and females (2014–2019 APC = −3.39, Fig 4G). This finding contrasts starkly with the global ASIR decline (S5 Fig). India shows an unstable “fluctuating upward” trend stemming from an overall increasing trend in ASIR for males and a decreasing trend in ASIR for females ((S6 Fig). China’s ASPR mirrored the ASIR trajectory, with critical turning points in 1995, 2000, 2005, 2015, and 2019. The period of increase occurred in 1995−2000 (Fig 4B), which is consistent with ASIR. However, males experienced a second period of increase (2010–2015 APC = + 0.17) (Fig 4E). The remaining periods were all periods of decline, with the most significant declines occurring in males (2015–2018 APC = −1.81) (Fig 4E) and females (2014–2019 APC = −3.00) (Fig 4F). The trend of China’s ASYR (Fig 4C) was similar to that observed in China’s ASPR. Global ASPR vs. ASYR trends were similar to those observed in global ASIR (S5 Fig). The trends in ASPR and ASYR in India were similar to those observed in ASIR in India (S6 Fig).

### AAPCs of incidence, prevalence and YLDs for VFs in China, India and throughout the world

Table 2 illustrates the AAPCs in the incidence, prevalence, and YLDs of VFs caused by MVRI over three decades in China. Between 1990 and 2021, China demonstrated age-standardized AAPCs of −0.478 (95% CI: −0.531, −0.426) for incidence, −0.178 (95% CI: −0.234, −0.121) for prevalence, and −0.200 (95% CI: −0.260, −0.139) for YLDs (Table 2). Notably, male cohorts exhibited consistently elevated AAPCs across all three metrics compared to female counterparts (Table 2). This gender-specific divergence in epidemiological trajectories was not geographically isolated; both the global and Indian datasets revealed analogous patterns, with male subjects systematically demonstrating higher AAPC magnitudes than female subjects.

**Table 2:** Joinpoint regression analysis: trends in age-standardized incidence, prevalence, YLDs rates (per 100,000 persons) among both sexes, males, and females in China, India and globally,1990–2021.

Region	Gender	Incidence, AAPC (95% CI)	Pravelance, AAPC (95% CI)	YLDs, AAPC (95% CI)
China	Both	−0.478(−0.531,-0.426)	−0.178(−0.234,-0.121)	−0.200(−0.260,-0.139)
	Male	−0.145(−0.216,-0.073)	0.099 (0.018,0.18)	−0.086(−0.016,0.188)
	Female	−1.241(−1.339,-1.144)	−0.709(−0.813,-0.604)	−0.790(−1.021,-0.559)
Global	Both	−1.839(−1.869,-1.808)	−2.389(−2.422,-2.355)	−2.389(−2.423,-2.356)
	Male	−1.678(−1.695,-1.66)	−2.261(−2.29,-2.233)	−2.260(−2.287,-2.233)
	Female	−2.144(−2.193,-2.096)	−2.588(−2.618,-2.558)	−2.595(−2.623,-2.568)
India	Both	−0.013(−0.054,0.028)	0.065 (0.017,0.112)	0.064 (0.019,0.109)
	Male	0.274(−0.054,0.028)	0.289 (0.241,0.336)	0.295(0.254,0.336)
	Female	−0.518(0.23,0.317)	−0.260(−0.33,-0.19)	−0.276(−0.353,-0.198)

### The incidence of VFs attributable to MVRI in China was analyzed by using the APC model

**Fig 5:**
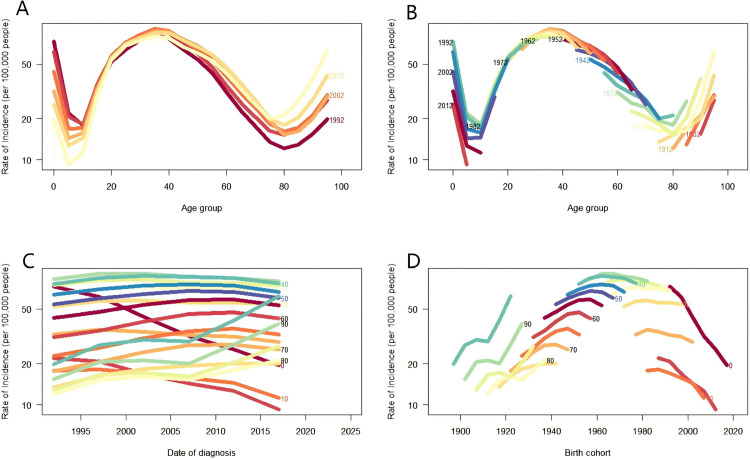
Age, period, and cohort effects on the incidence of VFs in China. (A) ASIR of VFs in different periods. Each line connects ASIR of 5 years. (B) ASIR of VFs in different birth cohorts. Each line connects age-specific incidence rates of a 5-year birth cohort. (C) Period-specific incidence rates for VFs in different age cohorts. Each line connects a 5-year age-specific incidence rates for the age cohort. (D) Birth cohort-specific incidence rates for VFs in different age groups. Each line connects a birth cohort-specific incidence rates for a 5-year age group. ASIR: age-standardized incidence rates.

Fig 5A presents the temporal trajectory of the incidence of VFs across age-stratified cohorts in China for 1992, 1997, 2002, 2007, 2012, and 2017. A sharp decline in incidence was observed among individuals aged 0–15 years, whereas the 15–40 and ≥60 cohorts experienced a notable increase that peaked in the 30–40 age bracket. Fig 5B presents the heterogeneity in the age-specific incidence patterns among different birth cohorts, with the consistently highest incidence at approximately 40 years of age. Fig 5C shows a universal upward trend in incidence across all adult age groups over time juxtaposed with a marginal decline in the < 15-year-old cohort (Fig 5D). The above trends were verified by cohort analysis, which showed that the late-born cohort had a greater prevalence than the early-born cohort after >15 years of age that increased with age. S7 Fig and S8 Fig illustrates analogous patterns for the prevalence and YLDs rate characterized by a bimodal distribution, with higher burdens being observed in elderly populations and comparatively lower rates being observed in later-born cohorts.

## Discussion

The evolutionary trajectory of the disease burden of motor vehicle-related VFs in China in 1990–2021 demonstrates a distinctive paradox within the global public health panorama and markedly contrasts with the significant decline observed in high-SDI regions (such as Sweden and the UK), where sustained traffic safety improvements have driven a 48.8% reduction in ASIR and a 22% reduction in incident cases over the same time period. Under the dual pressures of a motor vehicle fleet that surpassed 395 million and an aging population rate that escalated to 18.9% China demonstrated epidemiologic divergence with a 14.1% decline in ASIR but a 10.3% reverse increase in new cases. This epidemiological heterogeneity is not an isolated phenomenon; specifically, although India maintained ASIR stability at 3.22 per 100,000, it had a 77.2% increase in absolute cases, thus demonstrating systemic challenges inherent to rapid motorization [19]. In China, this was also evident as tech protection lagged behind risk growth due to insufficient social‒policy interaction [20]. Unlike the singular demographic-driven disease patterns observed in high-SDI or high-income countries, such as those in Western Europe, China’s situation represents the first documented instance where a middle-income country has experienced the compound epidemiological consequences of concurrent motorization and population aging. This unique phenomenon serves as a cautionary tale in for international public health [21]. Our findings are derived from population-level GBD estimates rather than single-center trauma registry data. Therefore, secular changes in local demographics, coding practices, trauma system organization, and injury-severity markers observed in specific settings (e.g., a single city over two decades) may not be fully mirrored in these aggregated estimates.

Changes in the incidence of MVRI leading to VFs and YLDs in China exhibit complex and variable characteristics with some correlations between the overall population and gender differences; however, each demonstrates different characteristics. An in-depth examination of the sex-stratified data reveals that in 2021, ASIR in China was 2.5 times greater in males (6.11 per 100,000) than in females (2.43 per 100,000). The deeper implications of this difference illustrate the gender differences in occupational risk in China during the process of industrialization [22]. The male incidence rate peaks at 30–34 years of age, which is closely related to the fact that young men are more likely to be exposed to occupational exposure (such as being freight drivers) and high-risk driving behavior (such as speeding, driving while fatigued, and driving under the influence of alcohol). This finding is in line with the general pattern of occupational risk shifting to young men in the early stages of motorization [23]. In contrast, the peak incidence in women aged 50–54 years is biomechanically coupled with a sudden drop in bone density after menopause [24,25]. Joinpoint regression analyses further reveal a significant increase in the incidence in men from 1995 to 2000 (APC=+2.14), whereas in women, there is a flat fluctuation during the same period (APC=+0.72). This dynamic change in gender stratification reflects not only the cumulative effect of occupational exposure but also the mismatch between biological vulnerability and social protection policies [26]. A significant decrease in incidence in China of −2.28 occurred in 2015–2019 and was not significant compared with the global trend of a significant decrease in all periods. This discrepancy reflects the rare phenomenon of “risk shifting” in industrialized countries. China, which is currently in a phase of rapid economic transition, must identify a balance between industrial expansion and both safety and security, whereas high-SDI regions have achieved risk reduction through technological upgrades [20]. Notably, the present estimates do not provide clinical granularity (e.g., fracture morphology and level, presence and severity of spinal cord injury, or standardized injury-severity indicators), which may partly explain differences from long-term observations in individual trauma systems.

The age distribution profile further illustrates the nature of the disease burden in China, where the triumphs of technological interventions stand in stark contrast to the persistent challenges posed by an aging population. Comparative demographic pyramid analyses between 1990 and 2021 revealed an 84.6% reduction in under-5 cases (9,238–1,424) that was validated by APC modeling and was attributable to China’s 2020 legislative mandate for child safety seats. In contrast, the incidence rate in the 60 + age group is gradually increasing. The use of the age effect shows that the incidence rate in 2020 increased in the older age group and created a “dual spectrum” of disease in parallel with osteoporosis [27]. This dichotomy of success in protecting children but not the elderly population is consistent with the trend of decreasing morbidity in children and increasing morbidity in the elderly population. Biomechanical studies have revealed that the underlying mechanism is age-related cumulative bone loss in people over 60 years of age (56% reduction in bone density compared with young people and aggravated with age) that alters the distribution of loads on osteoporotic trabeculae and results in a marked reduction in the load-bearing capacity of the vertebral bones of elderly individuals compared with young people. This scenario likely results in the scenario that low-impact crashes will be converted to fragility fractures [25,28]. Empirical studies have demonstrated that minor protective oversights, such as poorly designed bus seats and a lack of nonslip handrails, can increase the risk of VFs in elderly people on bumpy roads and during emergency braking [29]. This situation is more prominent in China, where protective oversights such as extremely low passenger seat belt use are prevalent and increase the likelihood of VFs among older passengers. However, the elderly population is characterized by a reduced perception of pain, and the lack of obvious clinical manifestations of occult VFs can lead to diagnostic and treatment gaps. Many elderly patients with undiagnosed VFs may not receive systematic diagnostic and therapeutic interventions, which increases their risk of secondary fracture chains [30]. All indications point to the urgent need to improve preventive measures in the elderly population. This complex trend, in conjunction with the general shift of occupational risk to young men in the early stages of motorization, has contributed to a slight decrease in the ASPR of only 5.8% in China, whereas the absolute number of cases has increased by 61.7%, which is in marked contrast to the global decline in the ASPR of 52.7% and the decline of 10.3% in the number of cases.

This difference in clinical outcomes ultimately translates into a considerable disease burden. Compared with the incidence rate, a lag in the YLDs of VFs in China was observed, thus indicating that the long-term impact of the disease is not effectively controlled. This is due mainly to the imbalance between the allocation of healthcare resources and the lack of timely treatment in China [31]. There is a scarcity of spine specialists in China; referral from county hospitals to tertiary hospitals takes an average of 6.2 hours, which misses the golden time for nerve repair. Cardiorespiratory insufficiency is common in elderly individuals, and when this condition is combined with high-energy trauma, it is prone to demonstrating complexity and variability and may require intensive care, thus resulting in high costs and forcing some patients to abandon treatment; this phenomenon is especially pronounced in low-income groups [32]. Through the use of advanced technologies such as robotics in combination with novel materials, it effectively reduces the risk of secondary neurological injuries and improves postoperative function. Recovery rates have increased [33], which resulted in a 52.4% decrease in YLDs worldwide between 1990 and 2021. Crawford N et al. reported that the use of robots in spine surgery can improve the accuracy of screw placement, reduce the risk of complications, and improve the overall surgical outcome [33]. Yingjie Wang et al. showed that novel materials such as LDH-modified PMMA bone cements can improve the overall outcome of spine surgeries through the modulation of osteogenic signaling pathways, such as p38 MAPK, ERK and MAPK, and by modulating the signaling pathway of osteogenesis (p38 MAPK, ERK/MAPK, FGF, and TGF-β) to accelerate osseointegration. Furthermore, its anti-inflammatory properties can reduce oxidative stress in the spinal cord compression microenvironment [34]. Despite the increased prevalence of vertebroplasty in China, county medical centers are still faced with a scarcity of spine specialists, a lag in medical technology, and inefficient transport. This multifaceted dilemma has resulted in a failure to effectively reduce YLDs in China over a 30-year time period. India’s YLDs are similar to those of China, and the shortcomings of its healthcare system serve as a warning to China: most injured individuals in rural areas do not reach trauma centers within one hour, and there is a severe shortage of spine specialists. Increasing the number of spine specialists in county hospitals and upgrading technology will effectively reduce YLDs, suggesting that optimizing resource allocation is significantly cost-effective [31].

The complexity of the burden of VFs in China stems from the interaction of multiple factors. High-SDI regions demonstrate significantly reduced traffic accidents through seatbelt regulations, the popularization of electronic stability control (ESC) technology and social collaboration. For example, Sweden has achieved a significant reduction in traffic accidents through a tripartite collaboration of technology, systems and society [35,36]. In China, however, there are problems such as lax enforcement of drunk driving laws in rural areas, low ESC coverage, and an imbalance of medical facilities between urban and rural areas. The comparison reveals that pure technology transplantation has limitations, and a multidimensional intervention system should be constructed to buffer the compound pressure of aging and motorization.

Based on the abovementioned results, we present several targeted observations. First, the screening of high-risk groups, the incorporation of bone density testing into the annual review of motor vehicle driver licenses, and the limitation of the driving time of high-risk individuals are recommended [37]. In addition, increased awareness of osteoporosis prevention in the elderly is necessary, as is protection for the elderly population. Moreover, traffic safety publicity and monitoring should be strengthened to increase citizens’ awareness of traffic safety. Furthermore, stricter traffic safety regulations should be enacted, along with the control of accident-prone roads; additionally, dangerous behaviors such as speeding and drunk driving should be investigated. The tertiary hospital-county medical community link and the prehospital grading system based on 5G + AI should be strengthened to optimize referral pathways and reduce patient retention time. Moreover, China may benefit from international collaboration to adopt advanced technologies and materials. Finally, the country must increase medical investments, reduce patients’ out-of-pocket medical expenses, and enhance their willingness to seek medical treatment. To comprehensively implement these measures, scientific breakthroughs are necessary, and policymakers must translate evidence into action to reshape public health resilience. Future work should integrate higher-resolution clinical or registry data to characterize vertebral fracture subtypes and levels, concomitant spinal cord injury, and injury-severity metrics, thereby improving interpretability and supporting more tailored prevention and care pathways.

## Conclusions

This study comprehensively analyzed the trends in VFs due to MVRI in China, India and globally in terms of gender and age during 1990–2021 using GBD 2021 database. A synergistic decline in the incidence, prevalence and number of YLDs and ASIR, ASPR and ASYR of VFs was achieved globally. China and India are experiencing greater disease burdens. These countries need to optimize the allocation of healthcare resources according to local conditions and improve access to spine specialties to cope with the compounded disease burden of motorization and aging. This study underscores the critical importance of technological innovation, adaptive policymaking, and multisectoral collaboration in alleviating the burden of traumatic fractures in China while offering valuable insights for formulating stratified intervention strategies across regions with different SDI levels.

## Limitations

This study has several limitations. First, geographic heterogeneity may lead to impaired data completeness of the GBD, such as registration gaps in the primary health information system in geographically remote regions of China. Second, the GBD definition of fracture is descriptive; thus, we were unable to assess the outcomes of fractures of varying severity. In addition, the fixed-effects parameter setting of DisMod-MR may introduce measurement error. It is recommended that future studies conduct comparative analyses using multiple models to reduce reliance on a single model’s output and improve the reliability of the estimation.

## Supporting information

S1 Checklist S_PLOS_Human_Participants_Research_Checklist_2025. (PDF)

S1 Fig Age-specific numbers and age-standardized incidence and prevalence rates of MVRI leading to VF in Global in 1990 and 2021. (A) Age-specific incidence numbers in 1990. (B) Age-specific incidence numbers in 2021. (C) Age-specific prevalence numbers in 1990. (D) Age-specific prevalence numbers in 2021. (E) The age-standardized incidence rate in 1990. (F) The age-standardized incidence rate in 2021. (G) Age-standardized prevalence rates in 1990. (H) The age-standardized prevalence rate in 2021.
(TIF)

S2 Fig Age-specific numbers and age-standardized incidence and prevalence rates of MVRI leading to VF in India in 1990 and 2021. (A) Age-specific incidence numbers in 1990. (B) Age-specific incidence numbers in 2021. (C) Age-specific prevalence numbers in 1990. (D) Age-specific prevalence numbers in 2021. (E) The age-standardized incidence rate in 1990. (F) The age-standardized incidence rate in 2021. (G) Age-standardized prevalence rates in 1990. (H) The age-standardized prevalence rate in 2021.
(TIF)

S3 Fig Trends in All-Age Cases and Age-Standardized Incidence, Prevalence, and Years of Disabled Life Lost (YLDs) Rates for VFs by Sex in Global, 1990–2021 (A) Number and Rate of Incidence (B) Number and Rate of Prevalence (C) Number and Rate of YLDs. (TIF)

S4 Fig Trends in All-Age Cases and Age-Standardized Incidence, Prevalence, and Years of Disabled Life Lost (YLDs) Rates for VFs by Sex in India, 1990–2021 (A) Number and Rate of Incidence (B) Number and Rate of Prevalence (C) Number and Rate of YLDs. (TIF)

S5 Fig In Global from 1990 to 2021, Joinpoint regression analysis was applied to the ASIR, ASPR and ASYR of VFs due to MVRI among males, females and both sexes, with a significance level of p < 0.05. (TIF)

S6 Fig In India from 1990 to 2021, Joinpoint regression analysis was applied to the ASIR, ASPR and ASYR of VFs due to MVRI among males, females and both sexes, with a significance level of p < 0.05. (TIF)

S7 Fig Age, period, and cohort effects on the prevalence of VFs in China. (A) ASPRs of VFs in different periods. each line connects ASPRs of 5 years. (B) ASPRs of VFs in different birth cohorts. each line connects age-specific prevalence rates of a 5-year birth cohort. (C) Period-specific prevalence rates for VFs in different age cohorts. Each line connects a 5-year age-specific prevalence rate for the age cohort. (D) Birth cohort-specific prevalence rates for VFs in different age groups. Each line connects a birth cohort-specific prevalence rate for a 5-year age group. ASPR Age-standardized prevalence rate.
(TIF)

S8 Fig Age, period, and cohort effects on the YLDs of VFs in China. (A) ASYRs of VFs in different periods. each line connects ASYRs of 5 years. (B) ASYRs of VFs in different birth cohorts. each line connects age-specific YLDs rates of a 5-year birth cohort. (C) Period-specific YLDs rates for VFs in different age cohorts. Each line connects a 5-year age-specific YLDs rate for the age cohort. (D) Birth cohort-specific YLDs rates for VFs in different age groups. Each line connects a birth cohort-specific YLDs rate for a 5-year age group. ASYR Age-standardized YLDs rate.
(TIF)

## Abbreviations

AAPC: Average Annual Percentage Change
APC: Age-Period-Cohort
ASIR: Age-Standardized Incidence Rate
ASPR: Age-Standardized Prevalence Rate
ASR: Age-Standardized Rate
YLDs: Years Lived with Disability
ASYR: Age-Standardized YLDs Rate
CI: Confidence Interval
CIR: Crude Incidence Rate
CPR: Crude Prevalence Rate
CYR: Crude Years Lived with Disability Rate
EMS: Emergency Medical Services
ERK: Extracellular Signal-Regulated Kinase
ESC: Electronic Stability Control
FGF: Fibroblast Growth Factor
GBD: Global Burden of Disease
ICD: International Classification of Diseases
LDH: Layered Double Hydroxide
MAPK: Mitogen-Activated Protein Kinase
MCMC: Markov Chain Monte Carlo
MVRI: Motor Vehicle Road Injury
PMMA: Polymethyl Methacrylate
TGF: Transforming Growth Factor
UI: Uncertainty Interval
UK: United Kingdom
VF: Vertebral Fracture
WHO: World Health Organization
