# Supplementary figures and images for: The 30-year evolution of motor vehicle road injuries: Can the future come from the shadows?
Source: PLoS One. 2026 Mar 3;21(3):e0342257. doi: 10.1371/journal.pone.0342257 (PMC12956069; doi:10.1371/journal.pone.0342257)

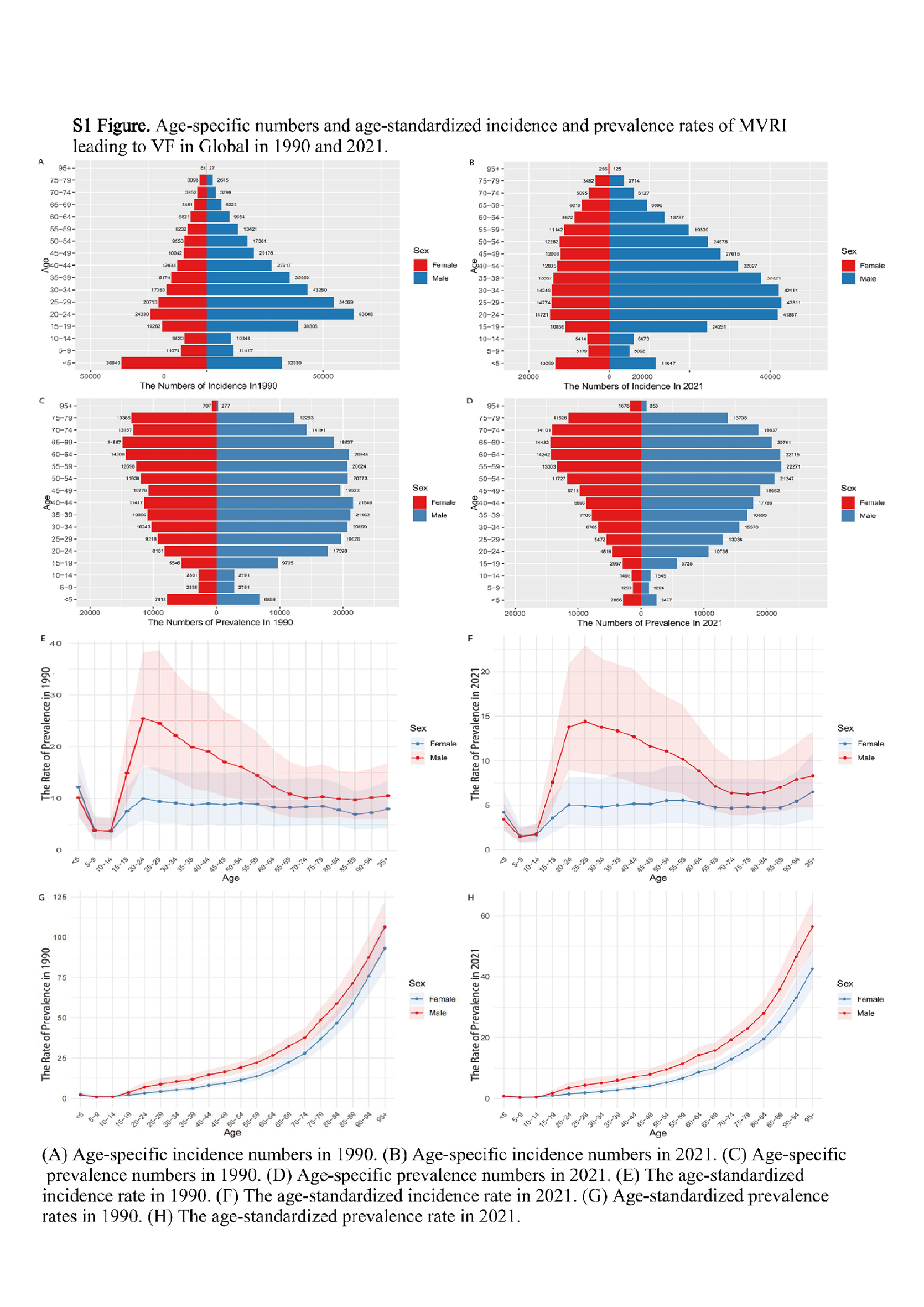

Supplement: S1 Fig — (A) Age-specific incidence numbers in 1990. (B) Age-specific incidence numbers in 2021. (C) Age-specific prevalence numbers in 1990. (D) Age-specific prevalence numbers in 2021. (E) The age-standardized incidence rate in 1990. (F) The age-standardized incidence rate in 2021. (G) Age-standardized prevalence rates in 1990. (H) The age-standardized prevalence rate in 2021. (TIF) [file pone.0342257.s002.tif]

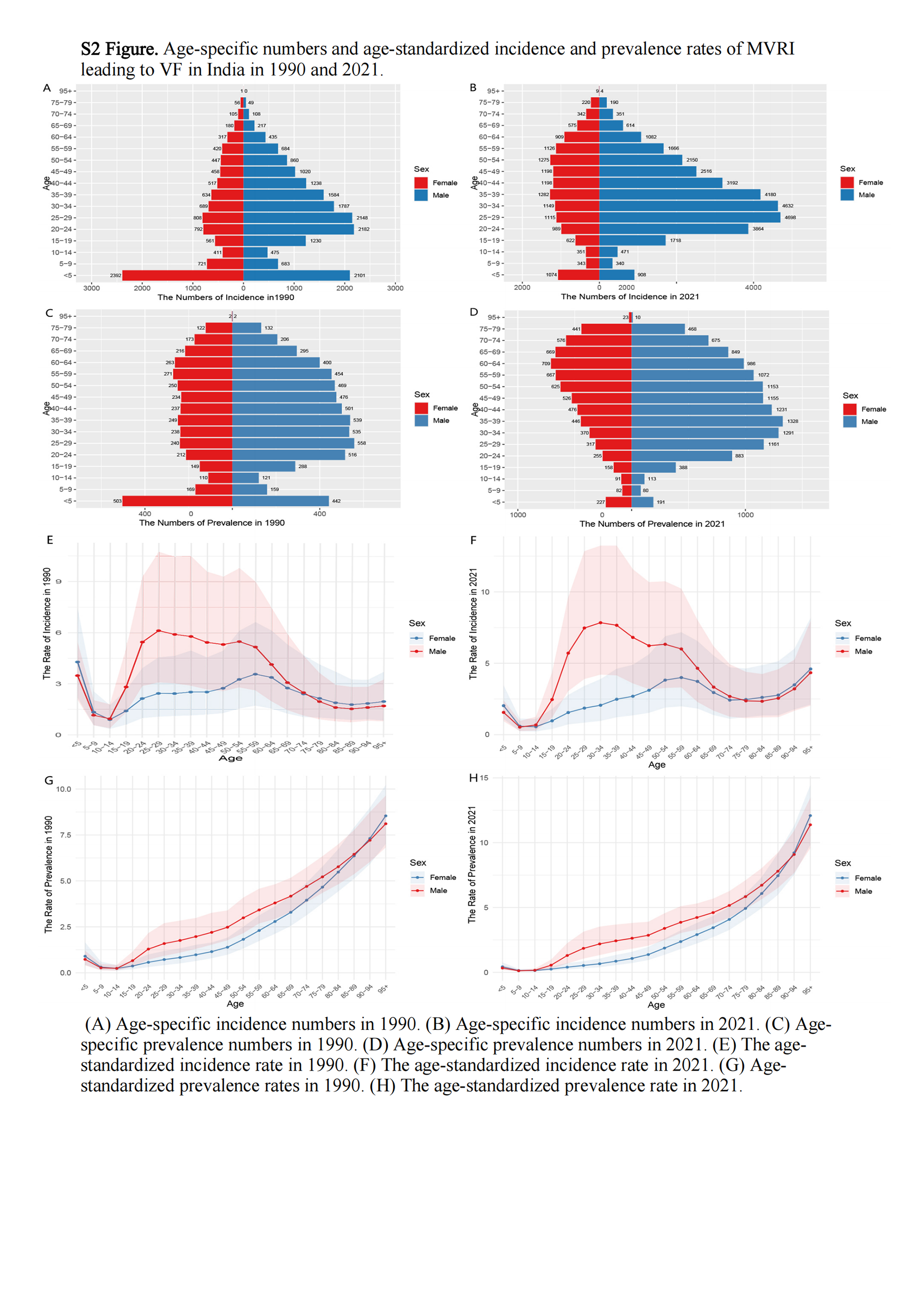

Supplement: S2 Fig — (A) Age-specific incidence numbers in 1990. (B) Age-specific incidence numbers in 2021. (C) Age-specific prevalence numbers in 1990. (D) Age-specific prevalence numbers in 2021. (E) The age-standardized incidence rate in 1990. (F) The age-standardized incidence rate in 2021. (G) Age-standardized prevalence rates in 1990. (H) The age-standardized prevalence rate in 2021. (TIF) [file pone.0342257.s003.tif]

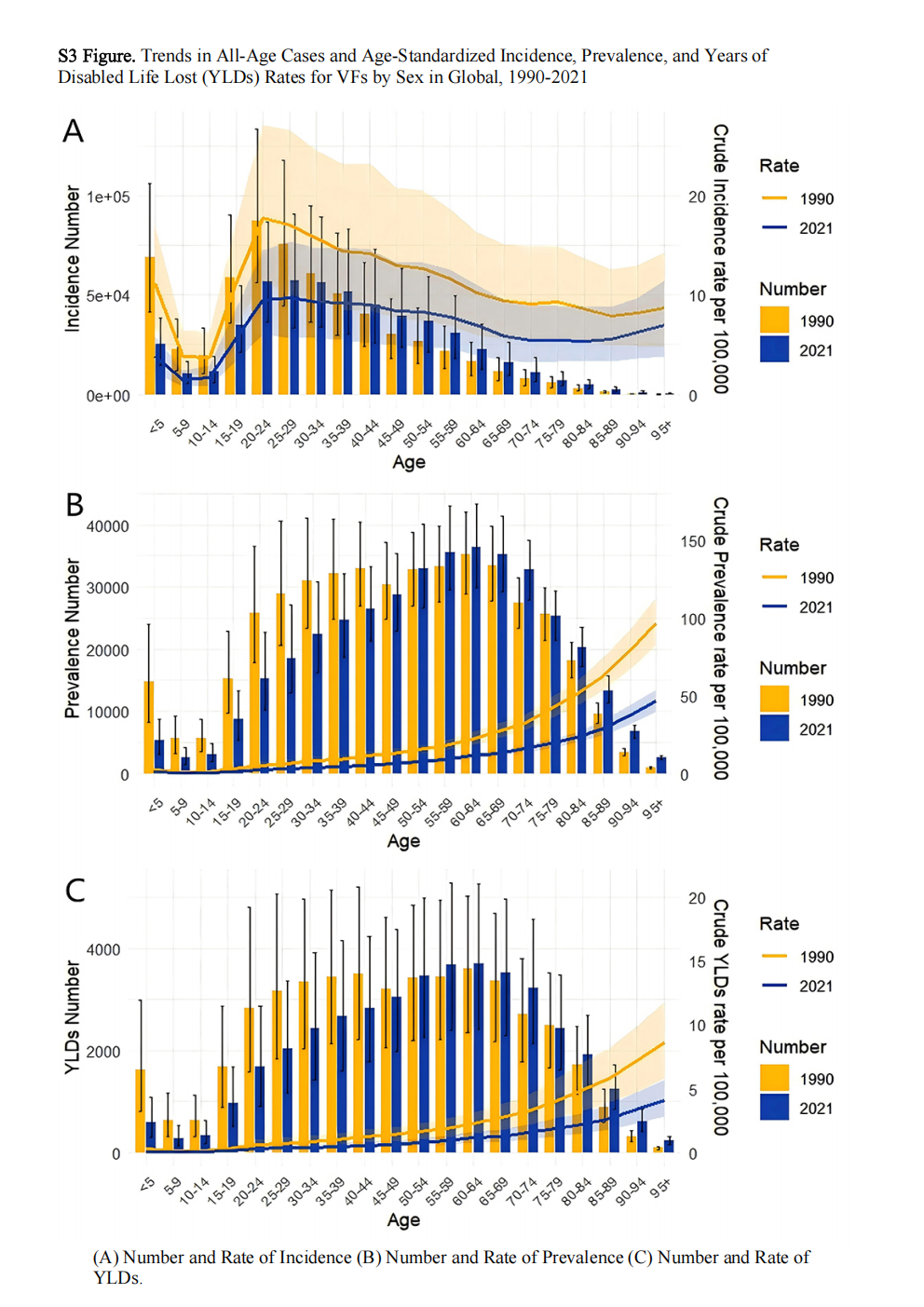

Supplement: S3 Fig — (TIF) [file pone.0342257.s004.tif]

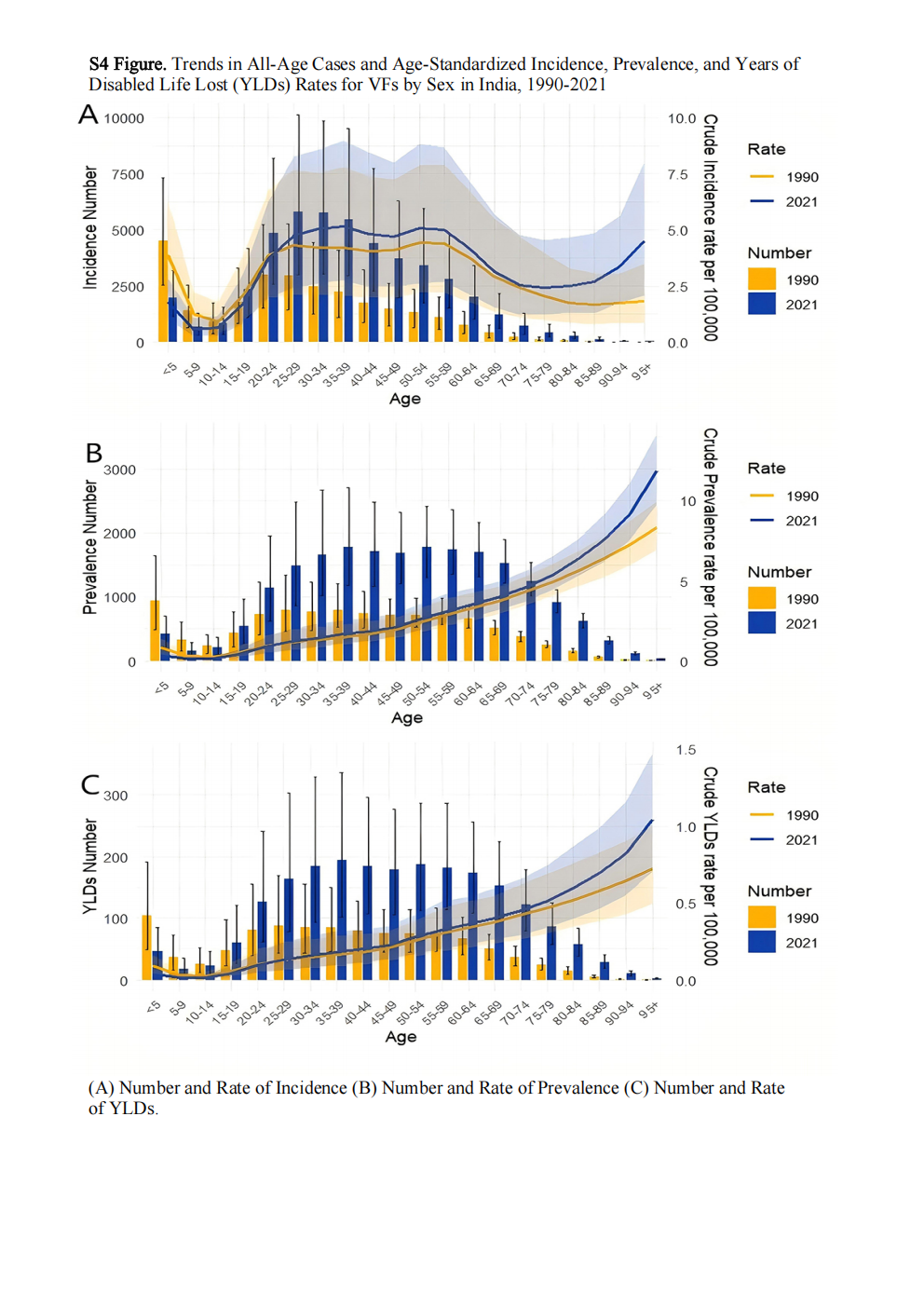

Supplement: S4 Fig — (TIF) [file pone.0342257.s005.tif]

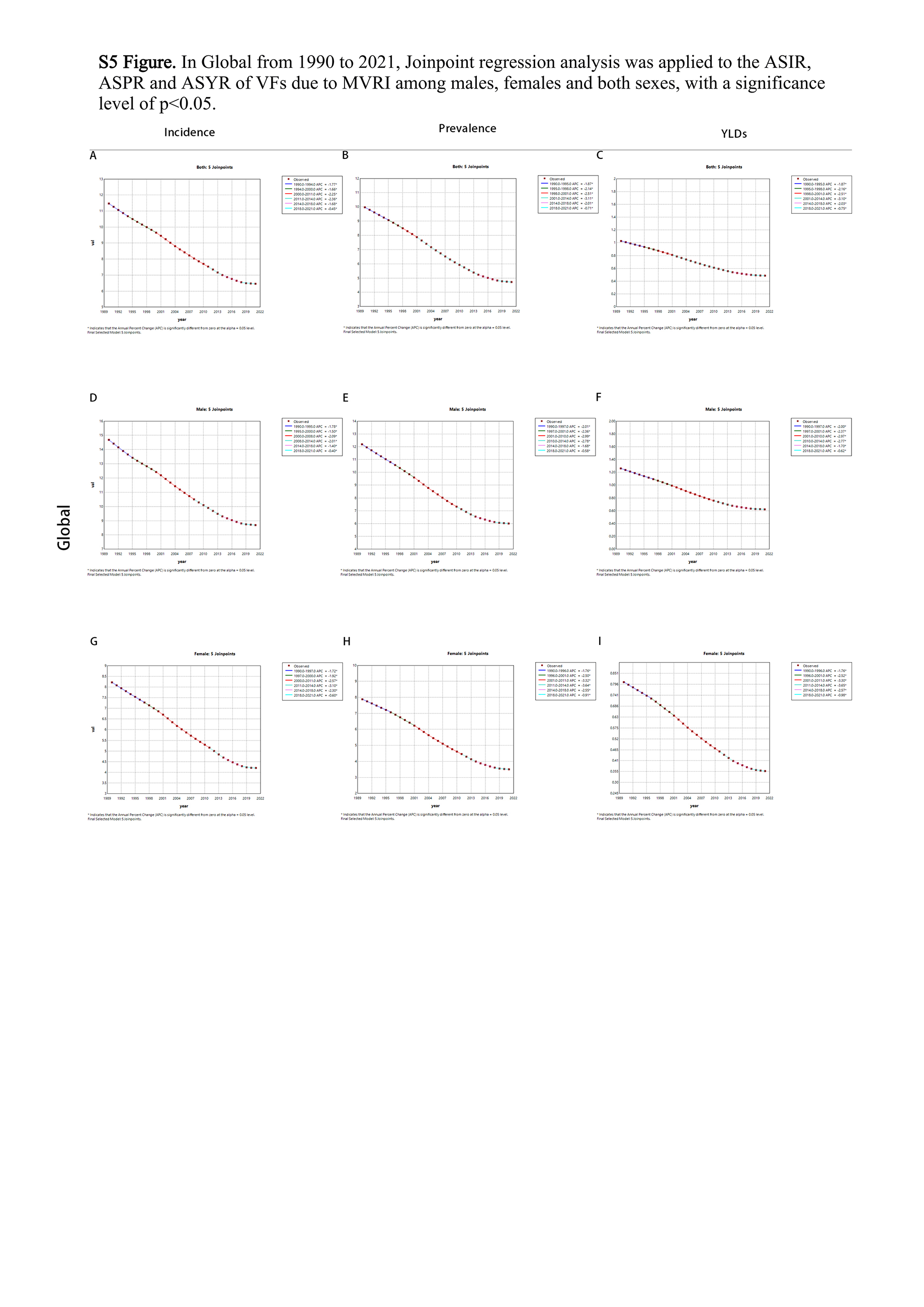

Supplement: S5 Fig — (TIF) [file pone.0342257.s006.tif]

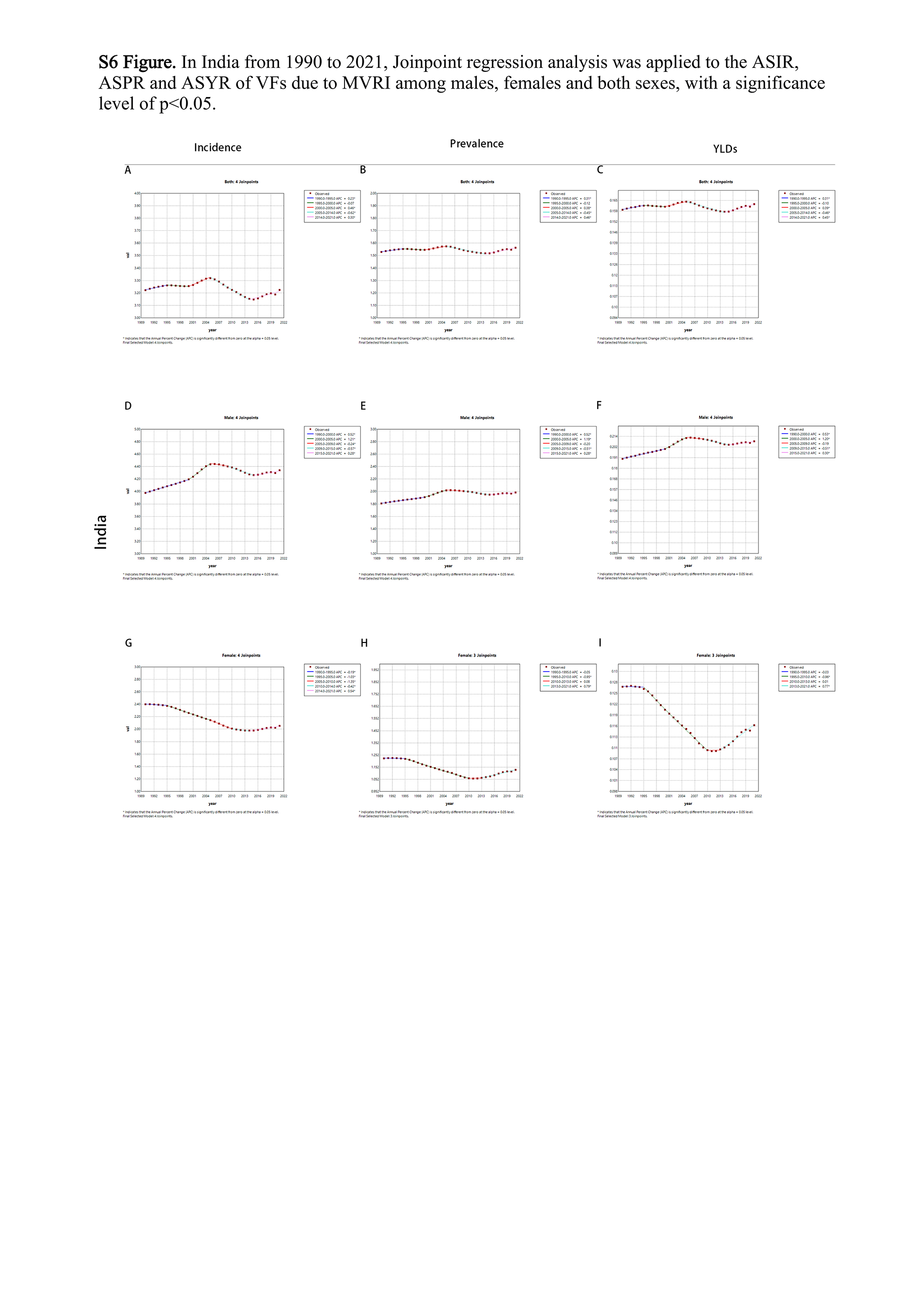

Supplement: S6 Fig — (TIF) [file pone.0342257.s007.tif]

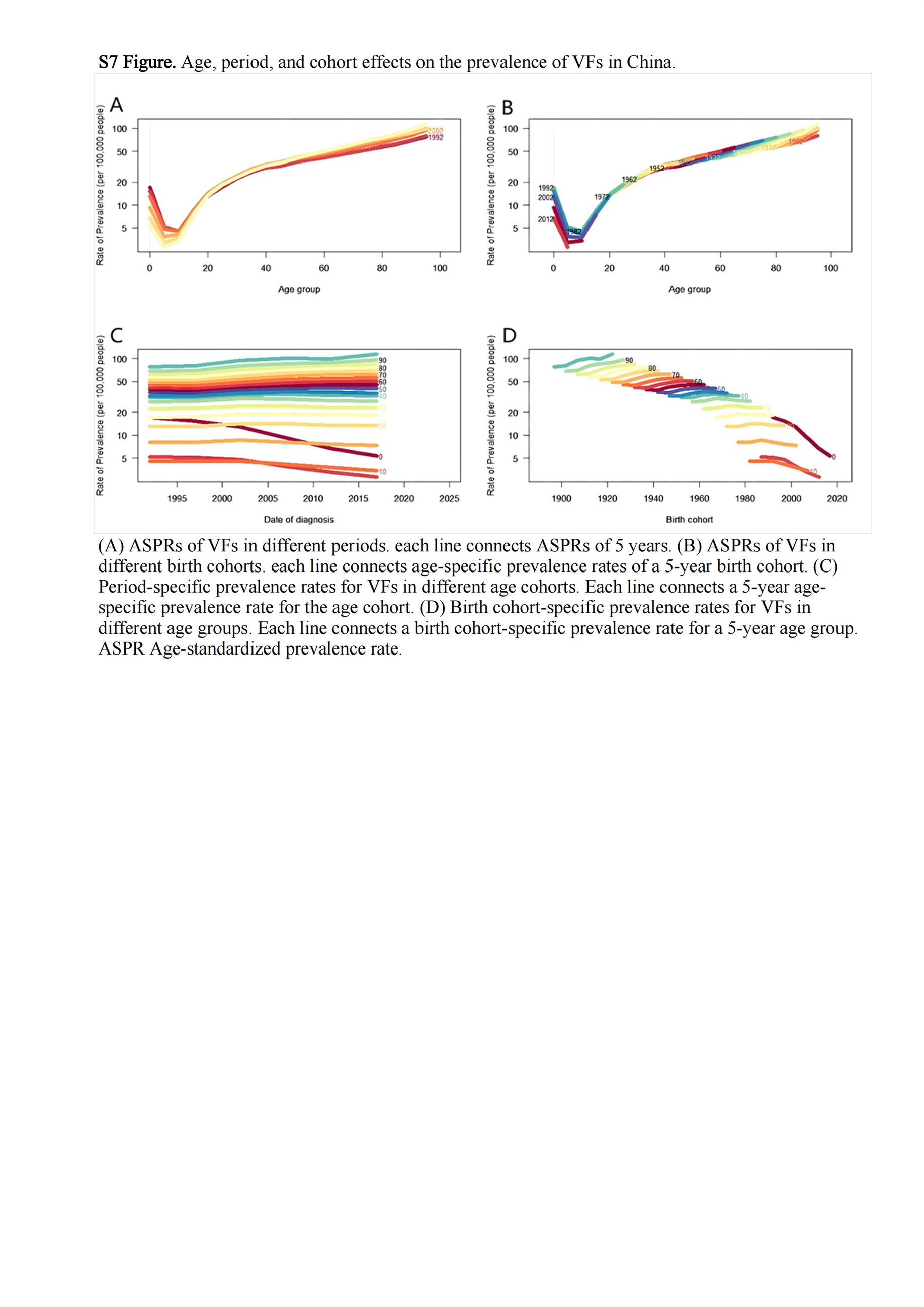

Supplement: S7 Fig — (A) ASPRs of VFs in different periods. each line connects ASPRs of 5 years. (B) ASPRs of VFs in different birth cohorts. each line connects age-specific prevalence rates of a 5-year birth cohort. (C) Period-specific prevalence rates for VFs in different age cohorts. Each line connects a 5-year age-specific prevalence rate for the age cohort. (D) Birth cohort-specific prevalence rates for VFs in different age groups. Each line connects a birth cohort-specific prevalence rate for a 5-year age group. ASPR Age-standardized prevalence rate. (TIF) [file pone.0342257.s008.tif]

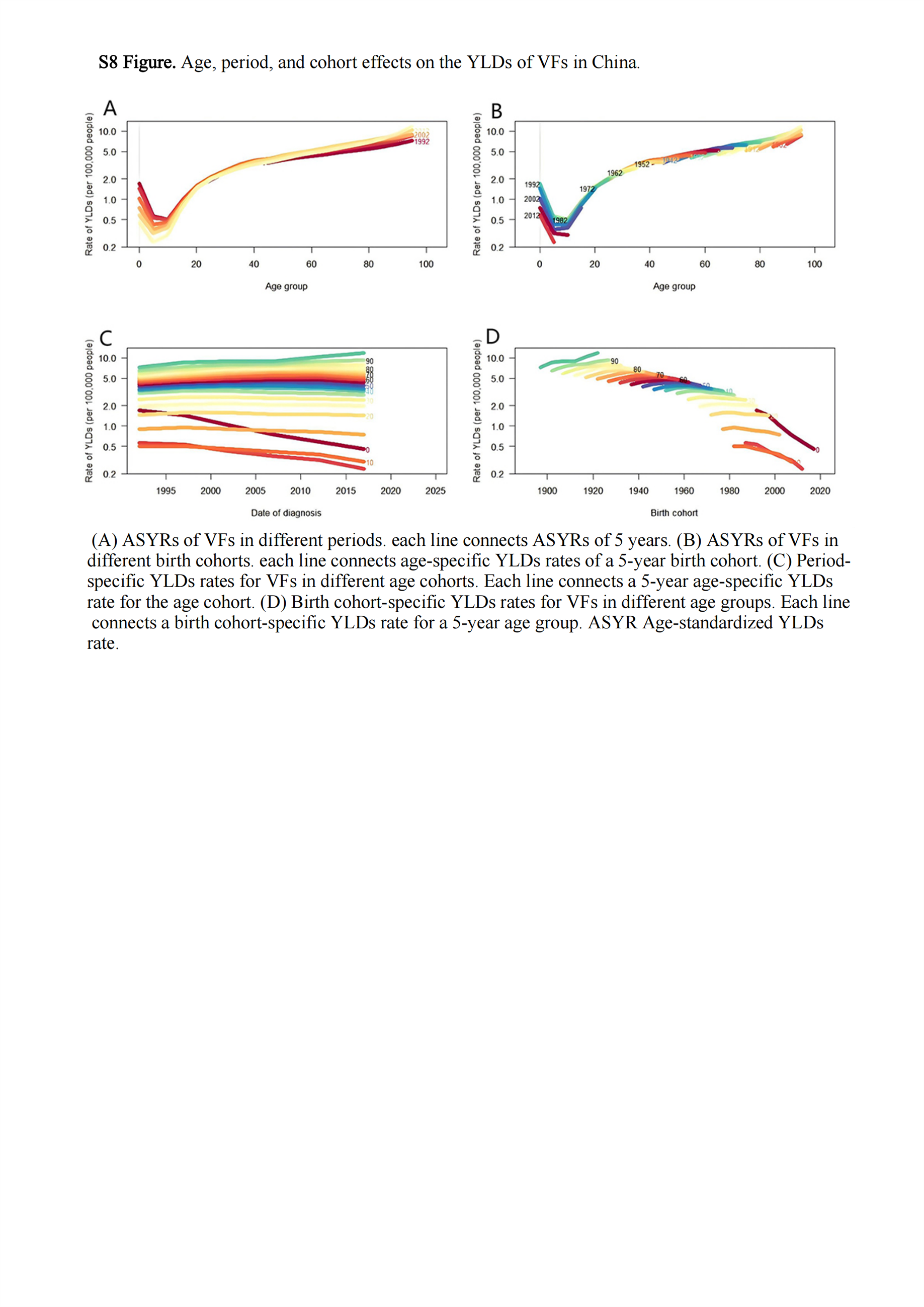

Supplement: S8 Fig — (A) ASYRs of VFs in different periods. each line connects ASYRs of 5 years. (B) ASYRs of VFs in different birth cohorts. each line connects age-specific YLDs rates of a 5-year birth cohort. (C) Period-specific YLDs rates for VFs in different age cohorts. Each line connects a 5-year age-specific YLDs rate for the age cohort. (D) Birth cohort-specific YLDs rates for VFs in different age groups. Each line connects a birth cohort-specific YLDs rate for a 5-year age group. ASYR Age-standardized YLDs rate. (TIF) [file pone.0342257.s009.tif]
